# Cefazolin and Ertapenem, a Synergistic Combination Used To Clear Persistent Staphylococcus aureus Bacteremia

**DOI:** 10.1128/AAC.01192-16

**Published:** 2016-10-21

**Authors:** George Sakoulas, Joshua Olson, Juwon Yim, Niedita B. Singh, Monika Kumaraswamy, Diana T. Quach, Michael J. Rybak, Joseph Pogliano, Victor Nizet

**Affiliations:** a University of California San Diego School of Medicine, La Jolla, California, USA; b Sharp Healthcare System, San Diego, California, USA; c Eugene Appelbaum College of Pharmacy and Health Sciences, Wayne State University, Detroit, Michigan, USA; d Department of Biological Sciences, University of California San Diego, La Jolla, California, USA; e Skaggs School of Pharmacy, University of California San Diego, La Jolla, California, USA

## Abstract

Ertapenem and cefazolin were used in combination to successfully clear refractory methicillin-susceptible Staphylococcus aureus (MSSA) bacteremia. In addition, recent work has demonstrated activity of combination therapy with beta-lactams from different classes against methicillin-resistant S. aureus (MRSA). The ertapenem-plus-cefazolin combination was evaluated for synergy *in vitro* and *in vivo* in a murine skin infection model using an index MSSA bloodstream isolate from a patient in whom persistent bacteremia was cleared with this combination and against a cadre of well-described research strains and clinical strains of MSSA and MRSA. Against the index MSSA bloodstream isolate, ertapenem and cefazolin showed synergy using both checkerboard (fractional inhibitory concentration [FIC] index = 0.375) and time-kill assays. Using a disk diffusion ertapenem potentiation assay, the MSSA isolate showed a cefazolin disk zone increased from 34 to 40 mm. *In vitro* pharmacokinetic/pharmacodynamic modeling at clinically relevant drug concentrations demonstrated bactericidal activity (>3 log_10_-CFU/ml reduction) of the combination but bacteriostatic activity of ether drug alone at 48 h. A disk diffusion potentiation assay showed that ertapenem increased the cefazolin zone of inhibition by >3 mm for 34/35 (97%) MSSA and 10/15 (67%) MRSA strains. A murine skin infection model of MSSA showed enhanced activity of cefazolin plus ertapenem compared to monotherapy with these agents. After successful use in clearance of MSSA bacteremia, the combination of ertapenem and cefazolin showed synergy against MSSA *in vitro* and *in vivo*. This combination may warrant consideration for future clinical study in MSSA bacteremia.

## INTRODUCTION

Staphylococcus aureus bacteremia is a common disease in a variety of host backgrounds, posing significant morbidity and mortality risks, especially in elderly patients and those hospitalized in the intensive care unit (1). Furthermore, bacteremia persistence for >3 or 4 days is a very strong predictor of mortality (2, 3). Due to the fact that the duration of bacteremia is about twice as long for methicillin-resistant S. aureus (MRSA) than for methicillin-susceptible S. aureus (MSSA), with a corresponding mortality that is also about 2-fold higher, much attention has been given to the development of salvage therapy for MRSA bacteremia (4, 5). However, there has been little evaluation of salvage therapy in the treatment of refractory MSSA bacteremia where a surgically addressable focus is not detected. Ertapenem (ETP)-plus-cefazolin (CZ) combination therapy was used to rapidly clear persistent MSSA bacteremia. An in-depth analysis of the synergy of the two drugs was performed at the level of the host innate immune system, as well as against a panel of clinical bloodstream isolates, demonstrating that ertapenem plus cefazolin requires clinical evaluation in the treatment of refractory MSSA bacteremia.

## MATERIALS AND METHODS

### Bacterial strains and *in vitro* antimicrobial susceptibility assays.

The index MSSA isolate for this study, rus276, was the original bloodstream isolate obtained from a patient with refractory MSSA bacteremia. It was examined using all the methods of susceptibility testing described below. CZ and ETP were purchased commercially (Sandoz Inc., Princeton, NJ, and Merck, Kenilworth, NJ, respectively). The well-characterized S. aureus strains SA113 (ATCC 35556) (MSSA) (6), MW2 (USA 400) (MRSA) (7), TCH 1516 (USA 300) (MRSA) (8), and Sanger 252 (USA200) (MRSA) (9) were evaluated for ETP and CZ synergy assays by checkerboard analysis in duplicate in Mueller-Hinton II (MHII) broth using a 10^5^-CFU/ml inoculum. In checkerboard assays, synergy was defined as a fractional inhibitory concentration (FIC) index of <0.5 (FIC index = MIC_ETP+CZ_/MIC_ETP_ + MIC_CZ+ETP_/MIC_CZ_).

Killing assays were performed in quadruplicate in brain heart infusion (BHI) broth with 0.06 mg/liter ETP and 0.25 mg/liter CZ, alone or in combination, using bacteria from an overnight culture diluted 1,000× to a starting inoculum of 6 or 7 log_10_ CFU/ml.

A group of 50 S. aureus isolates (35 MSSA and 15 MRSA) from a previously published clinical study (3) were evaluated for ETP and CZ synergy using disk diffusion. Disk diffusion synergy assays were performed using a modification of the Etest synergy assay described previously (10). A bacterial suspension of 0.5 McFarland standard (10^8^ CFU/ml) was streaked as a lawn on Mueller-Hinton agar (MHA) (2 plates/isolate). An ETP or CZ disk placed in the center of the plate was replaced with a new CZ disk or ETP disk. The diameter of the zone of inhibition was measured after incubation at 37°C for 24 h. For a subset of isolates, the disk diffusion test was repeated, where the second disk, placed after 1 h, was ETP. Synergy was defined as >3-mm increase in the zone size when sequential disks of different agents were used compared to the zone size with a single antimicrobial disk. This was established based on the performance of the index isolate, rus276, in the assay, which showed synergy by checkerboard analysis and kill curves. ETP and CZ MICs for the MSSA and MRSA strains were determined by CLSI broth microdilution methods (11). Assays on rus276 were performed twice on different days, and assays on the MSSA and MRSA clinical strains were performed once.

### Population analyses.

MSSA rus276 was grown to late stationary phase (18 to 20 h) in BHI or MHII broth and either plated directly onto BHI agar or MHA for the high-inoculum experiments or diluted 1,000× in fresh BHI or MHII broth and plated onto BHI or MHA plates for the low-inoculum condition. Ten-microliter aliquots were plated in duplicate with 10^0^ to 10^7^ dilution of each sample. The plates contained 2, 1, 0.5, 0.25, 0.125, 0.0625, and 0.0312 mg/liter of cefazolin and either 0, 0.03, or 0.06 mg/liter of ETP. Colonies were enumerated at 24 h, and the log_10_ CFU per milliliter was calculated for graphical depiction. The limit of detection of the assay was 2.0 log_10_ CFU/ml.

### Biofilm production.

The relative effects of sub-MICs of CZ and ETP, alone or in combination, on the ability of MSSA rus276 to produce biofilm were assessed using polystyrene adherence assays. The bacteria were grown for 16 to 20 h in BHI (late stationary phase) and diluted 1,000× in fresh Trypticase soy broth (TSB) supplemented with 1% glucose, and 100 μl/well was aliquoted onto sterile 96-well polystyrene enzyme-linked immunosorbent assay (ELISA) plates. Four conditions were tested, with 4 wells/condition: no antibiotic, 0.062 mg/liter CZ, 0.031 mg/liter ETP, or both ETP and CZ. The bacteria were grown for 24 h, and the optical density at 600 nm (OD_600_) was measured for each well to determine the density of bacterial growth. The medium was removed, the wells were washed 3 times with 200 μl sterile phosphate-buffered saline (PBS), and adherent cells were fixed at 65°C for 60 min and stained with 100 μl of crystal violet. Unbound stain was washed off with 200 μl of PBS, and adherent dye was extracted with 30% acetic acid for quantification by measurement of the OD_570_. Biofilm was expressed as the OD_570_/OD_600_ ratio to adjust for differences in bacterial growth under the different antibiotic conditions.

### One-compartment pharmacokinetic/pharmacodynamic in vitro models and pharmacokinetic analysis.

Cation-adjusted Mueller-Hinton broth (MHB) (Difco, Detroit, MI) was used for pharmacokinetic/pharmacodynamic (PK/PD) modeling and susceptibility testing. A 250-ml *in vitro* one-compartment model with inflow and outflow ports was utilized for human pharmacokinetic simulations. The model, prefilled with MHB, was inoculated with MSSA rus276 using bacterial lawns grown overnight. Antimicrobial agents were administered as a bolus at the defined frequency. Fresh medium was continuously infused and removed from the compartment, along with the drug, at a rate to simulate the half-life of the antimicrobial agent. In the models simulating combination therapy with CZ and ETP, the outflow rate was set up to simulate the half-life of CZ, and supplemental ETP was provided to compensate for excessive removal of ETP. The antimicrobial regimens simulated in this study were as follows: (i) 2 g CZ every 8 h (q8h) (*fC*_max_ [maximum free drug concentration], 31.1 μg/ml; half-life, 2 to 2.7 h) (12, 13); (ii) 0.5 g ETP q24h (*fC*_max_, 4.71 μg/ml; half-life, 4 h) (14); (iii) 2 g CZ q8h plus 0.5 g ETP q24h. All experiments were performed in duplicate to ensure reproducibility.

Pharmacokinetic samples were obtained at appropriate time points through the injection ports of the models and analyzed by bioassay as described previously to verify the attainment of the target pharmacokinetic parameters (15). Both CZ and ETP concentrations were measured via bioassays using Bacillus subtilis ATCC 6633 and Escherichia coli ATCC 25922 as the test organisms on antibiotic medium 11 (16, 17). The relevant pharmacokinetic parameters achieved were determined by the trapezoidal method using PK analyst software (version 1.10; MicroMath Scientific Software, Salt Lake City, UT).

Broth samples were obtained from each model at 0, 4, 8, 24, 32, and 48 h and plated on tryptic soy agar (Difco, Detroit, MI) after appropriate serial dilutions in 0.9% saline using an automatic spiral plater (Wasp; DW Scientific, West Yorkshire, England). The plates were incubated for 18 to 24 h at 37°C before colony enumeration with a laser colony counter (Scan 1200; Interscience Laboratories Inc., Woburn, MA). The remaining bacterial counts, which were reported in log_10_ CFU per milliliter, were plotted against each time point to determine the total reduction in the bacterial burden over 48 h. Bactericidal and bacteriostatic activities were defined as a ≥3 log_10_-CFU/ml and as a <3 log_10_-CFU/ml reduction in the bacterial colony count from the initial inoculum, respectively. The synergistic and additive activities of a combination were defined as an increase in killing of ≥2 log kill and <2 but >1 log kill in comparison to the most active single agent of the combination (18). Therapeutic enhancement of an antibiotic combination was defined as ≥2 log_10_-CFU/ml greater bacterial killing than the most active single agent when the most active agent resulted in >90% (1 log_10_) kill (19). Statistically significant differences were assessed using one-way analysis of variance (ANOVA) with Tukey's *post hoc* test.

### Fluorescence microscopy.

Exponential-phase cell cultures (OD_600_, ∼0.1) were treated with antibiotics (CZ and/or ETP) and grown at 37°C in a roller. Samples were collected for imaging after 3 h. Eight microliters of cells was added to 2 μl of dye mix consisting of 2.5 μM Sytox Green (Life Technologies, Eugene, OR), 10 μg ml^−1^ DAPI (4′,6-diamidino-2-phenylindole) (Life Technologies, Eugene, OR), and 20 μg ml^−1^ WGA-647 (Life Technologies, Eugene, OR) in 1× Tris base and transferred to an agarose pad (10% MHB, 1% agarose). The exposure time of each wavelength was maintained constant for all images. Microscopy was performed as previously described (20, 21). Quantification of the mean DAPI and Sytox Green intensities was performed using CellProfiler (22) as previously described (21).

### Human cathelicidin LL-37 susceptibility assays.

Human cathelicidin (LL-37) MIC susceptibility testing and killing assays were performed in RPMI medium supplemented with 10% LB medium as previously described (23, 24). Briefly, the MIC was determined, and killing assays were performed at 1/2 the MIC of LL-37 alone or combined with ETP and/or CZ. Killing assays were performed in quadruplicate.

### Neutrophil killing assays.

Neutrophils were freshly isolated from the blood of healthy donors using a PolyMorphPrep kit (Fresenius Kabi), and erythrocytes were lysed with sterile water as previously described (25). Bacteria were inoculated at a multiplicity of infection (MOI) of 100 with 5 × 10^5^ neutrophils in RPMI medium utilizing suspension culture plates (26). After incubation for 15 and 45 min at 37°C and 5% CO_2_, the cells were lysed with 0.025% Triton X-100, and the total bacteria remaining were enumerated on Luria agar plates. The percent bacterial survivals at 15 and 45 min of incubation were calculated based on the average number of CFU per milliliter noted for each strain at 15 min. The use of and procedures for these experiments were approved by the University of California San Diego Human Research Protections Program.

### Mouse infection model.

rus276 was grown overnight in LB broth and resuspended to approximately 10^7^ CFU/ml in sterile PBS, and 0.1 ml (10^6^ CFU) was injected subcutaneously into 25-g female CD1 mice. Four hours later, therapy was begun with either 0.5 mg CZ (20 mg/kg of body weight), 0.5 mg ETP (20 mg/kg), both CZ and ETP, or PBS (control) administered in a 0.1-ml volume intraperitoneally every 8 h for 3 doses. The doses were extrapolated based on prior doses established in laboratory animals (27). The animals were sacrificed 2 to 3 h after the last dose; visible lesions were excised, weighed in sterile vials, and homogenized in sterile PBS; and serial 10-fold dilutions were plated for enumeration 24 h later. The results were expressed as CFU of bacteria per gram of tissue. The results represent a combination of 2 experiments done on different days, with 6 or 7 mice/group in each of the 2 experiments. Animals were maintained in accordance with the American Association for Accreditation of Laboratory Animal Care Criteria, and the above-described studies were approved by the Animal Care and Use Committee (IACUC) of the University of California, San Diego.

## RESULTS

### Case description.

An 80-year-old female bed-bound 3-year nursing home resident was admitted with shortness of breath and weakness. Her relevant history included diabetes mellitus type 2, obesity, congestive heart failure, pulmonary thromboembolic disease, and chronic back pain for over 3 years. In the emergency room, she was tachypneic, with a temperature of 37°C, a respiratory rate of 30, a heart rate of 130, and 97% on oxygen on a nonrebreather mask. Laboratory data showed a peripheral white blood cell (WBC) count of 11,000/mm^3^ (89% neutrophils), hemoglobin at 10.4 mg/dl, a platelet count of 222,000/mm^3^, and creatinine at 0.9 mg/dl. A chest X-ray showed possible left lower lobe pneumonia. Blood cultures grew Gram-positive cocci in clusters, and 600 mg ceftaroline intravenously (i.v.) q8h was initiated. C-reactive protein (CRP) was 308 mg/liter (normal, <5 mg/liter). In 72 h, the identification of the blood cultures showed MSSA (isolate rus276, used in subsequent laboratory studies), with one bottle also growing Staphylococcus epidermidis, which was deemed a contaminant. Antibiotic therapy was switched to 2 g CZ i.v. q8h due to a recent history of severe rash with penicillins. Despite this, the blood cultures remained positive. Thorough imaging evaluation revealed multifocal pneumonia suggestive of septic pulmonary emboli, and echocardiography showed a normal ejection fraction (>50%), trace mitral regurgitation, left ventricular hypertrophy, right atrial pressures of 10 to 20 mm Hg, and no evidence of vegetation. Body habitus precluded magnetic resonance imaging (MRI), but a Tc-99 bone scan showed evidence of osteomyelitis at the T10 to T12 spine. Given that she was not a surgical candidate for any valvular heart surgery, a transesophageal echocardiogram (TEE) was deferred after discussions with the patient.

After 5 days of persistent MSSA bacteremia, 1 g ETP i.v. q24h was added to CZ, and daily blood cultures were monitored. The day after ETP was added, the blood cultures became negative and remained negative for 3 consecutive days, after which daily blood culture monitoring was discontinued. CRP was down to 115 mg/liter 13 days into antibiotic therapy. The patient was discharged to a subacute nursing facility to complete 2 weeks of combination therapy with 1 g ETP i.v. q24h plus 2 g CZ i.v. q8h, followed by an additional 4 weeks of CZ monotherapy. She remained free of bacteremia recurrence 90 days after therapy.

### Antibacterial susceptibility and killing.

**FIG 1:**
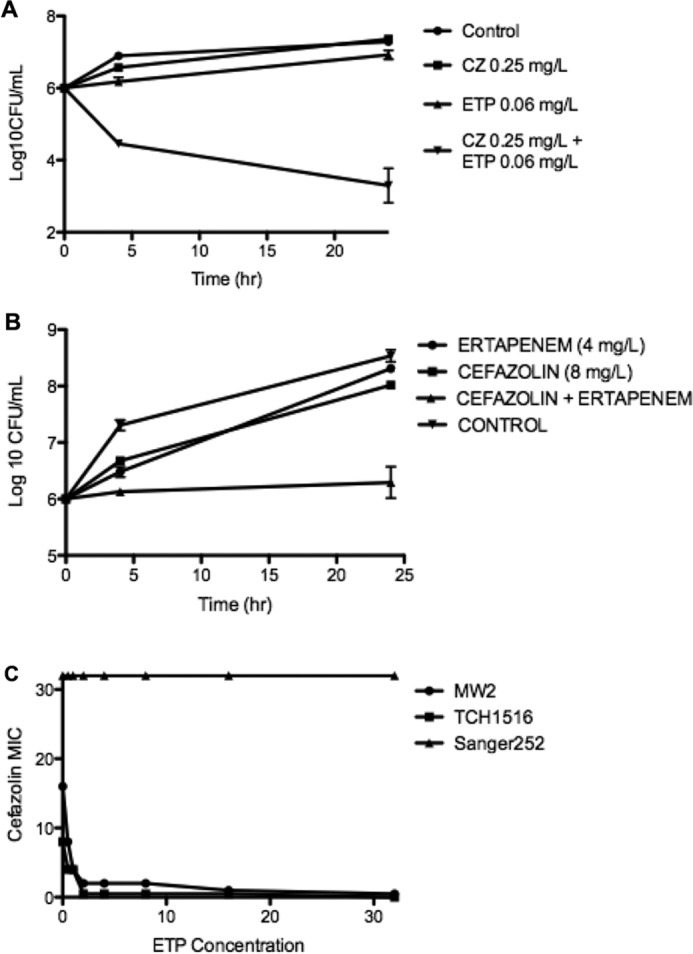
(A) Kill curves of the index MSSA isolate, rus276, employing 1/2 MIC of CZ or ETP alone or in combination. (B) Kill curve assays of MRSA MW2 containing ≤1/4 MIC of cefazolin, ertapenem, or both drugs. (C) Results of checkerboard analysis of susceptibilities to cefazolin MICs in media containing various concentrations of ertapenem for MRSA strains MW2, TCH 1516, and Sanger 252. The error bars indicate standard deviations.

The index isolate rus276 was evaluated for synergy between ETP (MIC, 0.125 mg/liter) and CZ (MIC, 0.5 mg/liter) using checkerboard assays, showing an FIC index of 0.25 plus 0.125, equal to 0.375. With 0.25 mg/liter CZ (1/2 MIC), the ETP MIC was reduced by 2 dilutions (0.25×) to 0.03 mg/liter; with 0.06 mg/liter ETP (1/2 MIC), the CZ MIC was reduced by 3 dilutions (0.125×) to 0.06 mg/liter. The isolate was then examined by killing assays employing ETP and CZ alone or in combination in BHI broth at 1/2 MIC (Fig. 1A). Clear synergy was demonstrated, where growth was seen in the presence of either antibiotic alone but nearly 3 log_10_-CFU/ml killing was seen with the two drugs in combination at these concentrations.

Given recent data showing that carbapenems and piperacillin-tazobactam exhibited synergistic killing of MRSA, checkerboard analysis between ETP and CZ was performed for MRSA strains Sanger 252 (USA200), TCH 1516 (USA 300), and MW2 (USA 400). Synergy was noted for TCH 1516 (FIC index = 0.140) and MW2 (FIC index = 0.05), but not Sanger 252, which showed indifference (FIC index = 2). Killing assays with CZ (8 mg/liter) and ETP (4 mg/liter) alone and in combination were performed with MW2, showing growth of the bacteria with antibiotic-free medium and each antibiotic alone but static activity with the two drugs together (see Fig. S1B in the supplemental material). Figure 1C shows the MICs of CZ in the presence of various concentrations of ETP.

### Fluorescence microscopy studies.

**FIG 2:**
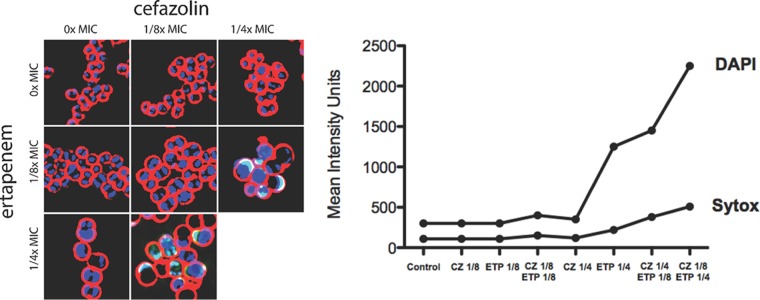
(Left) Microscopy comparing the effects of CZ or ETP alone and in combination on MSSA rus276 at sub-MICs. Sytox Green and DAPI stain uptake was used to measure cellular permeabilization. (Right) Quantification of DAPI and Sytox staining, reflective of increases in cellular permeability with combined ETP and CZ compared to either drug alone at concentrations relative to the MIC.

Given the above-mentioned results, it was deemed important to determine that the observed synergy was not simply the result of a higher concentration of cumulative beta-lactam in the combination drug experiments than of each drug alone. In other words, we needed to differentiate a potentiation of CZ activity by ETP above what would be observed by doubling the concentration of CZ alone. In order to do this, we used fluorescence microscopy to differentiate the killing phenotype of each drug alone or in combination at sub-MICs (Fig. 2A). Treatment of rus276 with sub-MIC levels (1/4 and 1/8 MIC) of each antibiotic alone resulted in slight swelling of the cells, continued cellular division, and little to no cell lysis. In contrast to treatment with 1/4 MIC of a single antibiotic, the addition of 1/8 MIC of the second antibiotic resulted in swelling of the cells, increase of lysis, and inhibition of cell division, observed as a single decondensed DNA nucleoid per cell. The difference in lysis between different antibiotic treatments was quantified by measuring the intensities of the cell permeability indicator Sytox Green, which is membrane impermeable, and the DNA dye DAPI, which increases in intensity when the membrane becomes more permeabilized (Fig. 2B). Of interest, ETP appeared to show greater lethal effects on bacterial cells than CZ, despite the clinically accepted superiority of CZ as an antistaphylococcal antibiotic.

### ETP reduces CZ heteroresistance.

**FIG 3:**
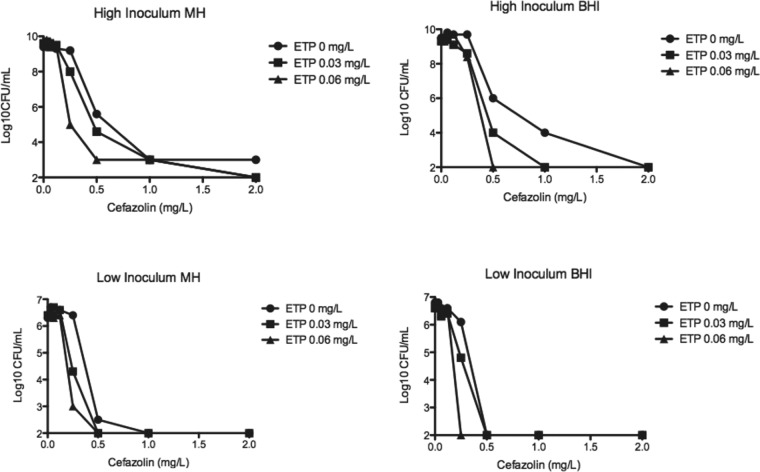
CZ population analyses performed on MSSA rus276 under low (10^6^ CFU/ml) and high (10^9^ CFU/ml) inocula using Mueller-Hinton II (MH) or BHI agar containing 1/2 and 1/4 MIC ETP. ETP consistently reduced CZ heteroresistance, particularly under high-inoculum conditions.

Given the reduced CZ MIC that has been previously described for some MSSA strains when using a high inoculum, we performed CZ population analyses for MSSA rus276 in BHI and MHA utilizing 0 mg/liter, 0.03 mg/liter, and 0.062 mg/liter ETP. The results are shown in Fig. 3, utilizing both high-inoculum (10^9^ CFU/ml) and low-inoculum (10^6^ CFU/ml) conditions. In both media, there was a consistent dose-dependent reduction in CZ heteroresistance with ETP, which was more pronounced under high-inoculum conditions.

### ETP attenuates biofilm formation induced by CZ.

Given the importance of the biofilm phenotype in the establishment and propagation of S. aureus endovascular infection, we examined the biofilm properties of MSSA rus276 in the presence or absence of 1/4 MIC ETP and CZ, alone and in combination. Consistent with prior studies of subinhibitory concentrations of beta-lactams and biofilm formation in S. aureus (28, 29), CZ actually enhanced biofilm formation. However, ETP alone or ETP plus CZ showed reduced biofilm formation compared to the control (see Fig. S1 in the supplemental material).

### Enhancement of innate immune killing.

**FIG 4:**
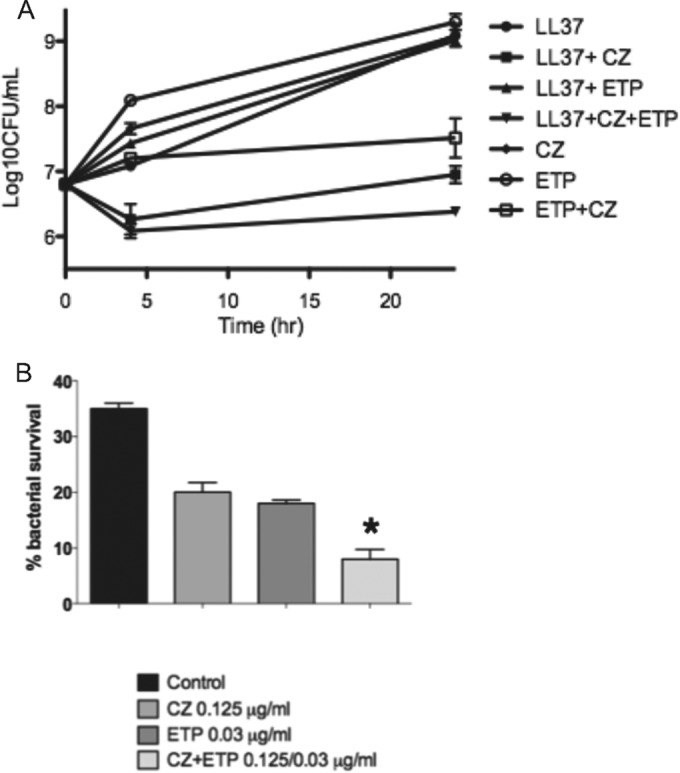
(A) Kill curve assays of MSSA rus276 using human cathelicidin LL-37, CZ, or ETP alone or in combination. (B) Neutrophil killing assays of MSSA rus276 after growth in antibiotic-free medium (control) or media containing CZ, ETP, or both drugs. The results are expressed as percentages of bacterial survival remaining after 90 min compared to the starting bacterial density (*, *P* < 0.05 versus the control). The error bars indicate standard deviations.

Killing assays were performed with CZ and ETP, alone and in combination, in the presence or absence of LL-37. The greatest killing was seen with the combination of the two antibiotics in the presence of LL-37 (Fig. 4A). Interestingly, no synergy was seen between ETP and LL-37, whereas CZ alone plus LL-37 showed greater killing than the combination CZ plus ETP. Bacteria were grown in the presence of each antibiotic alone or in combination and subjected to neutrophil killing assays. Significantly increased susceptibility to neutrophil killing at 90 min was seen with combination antibiotic pretreatment compared to no antibiotic or one antibiotic (*P* < 0.02; Mann-Whitney U test) (Fig. 4B).

### Pharmacokinetic and pharmacodynamic results.

**FIG 5:**
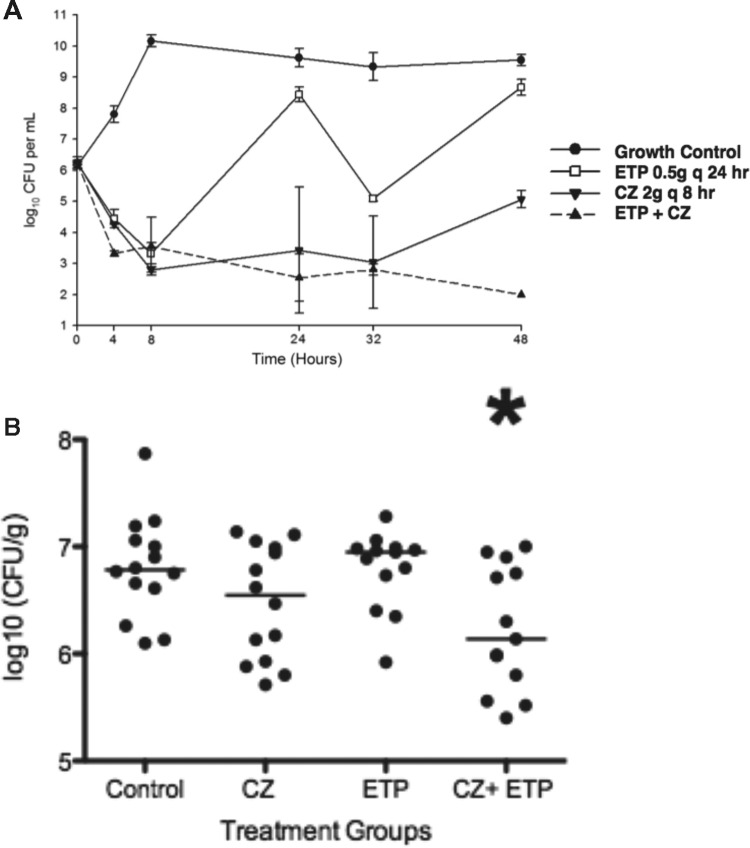
(A) Simulated pharmacokinetic/pharmacodynamic modeling of MSSA rus276 employing CZ and ETP alone or in combination at clinically relevant concentrations. Only the combination regimen of ETP plus CZ achieved bactericidal activity at 24 and 48 h, and CZ plus ETP showed significantly lower CFU at 48 h than the other groups (*P* < 0.01; one-way ANOVA with Tukey's *post hoc* test). The error bars indicate standard deviations. (B) Bacterial counts in excised skin tissue of mice treated with CZ, ETP, or both drugs versus no antibiotics (control). The horizontal bars indicate the medians of the groups. Only the combination treatment group (CZ+ETP) showed bacterial counts less than those with the control (*, *P* < 0.02; Mann-Whitney U test). Control versus CZ (*P* = 0.17) and control versus ETP (*P* = 0.87) were not statistically significant.

The average *fC*_max_ achieved in the models for CZ was 29.85 ± 0.18 μg/ml (target *fC*_max_, 31.1 μg/ml), and the average half-life was 2.25 ± 0.02 h (target half-life, 2 to 2.7 h). For ETP, an average *fC*_max_ of 5.11 ± 0.31 μg/ml (target *fC*_max_, 4.71 μg/ml) and half-life of 3.60 ± 0.001 h (target half-life, 3.8 h) were achieved. Against MSSA rus276, ETP was bacteriostatic at 8 h but regrew at 24 h, similar to drug-free growth control. CZ was initially bactericidal but started to regrow at 24 h and failed to maintain bactericidal activity at 48 h. Only a combination regimen of ETP plus CZ achieved bactericidal activity at 24 h and maintained it over 48 h (Fig. 5A). At 48 h, CZ plus ETP achieved therapeutic enhancement over CZ alone and was significantly better (*P* < 0.001) than the control, CZ, and ETP (one-way ANOVA with Tukey's *post hoc* test).

### Murine subcutaneous-infection model.

Treatment of a murine subcutaneous-infection model with CZ, ETP, or both drugs was evaluated by quantification of the bacterial inoculum per gram of tissue. The results are shown in Fig. 5B. Whereas CZ monotherapy trended toward a reduced bacterial inoculum compared to untreated animals at the dose provided (*P* = 0.17), only the CZ-plus-ETP combination regimen provided significant reduction in the number of CFU per gram compared to the control (*P* < 0.02; Mann-Whitney U test).

### Assessment of a clinical strain collection for ETP-plus-CZ synergy.

To further characterize the generalizability of the synergy between ETP and CZ against a larger set of clinical strains, we evaluated the disk diffusion method, where the first antibiotic disk was placed on a lawn of the test strain for 1 h and then removed and replaced with either the same disk or the other agent. Zone sizes were compared 24 h later. The index strain, rus276, showed an increase in CZ zone diameter from 34 to 40 mm when the ETP disk was replaced by a CZ disk compared to when a CZ disk was used alone.

**FIG 6:**
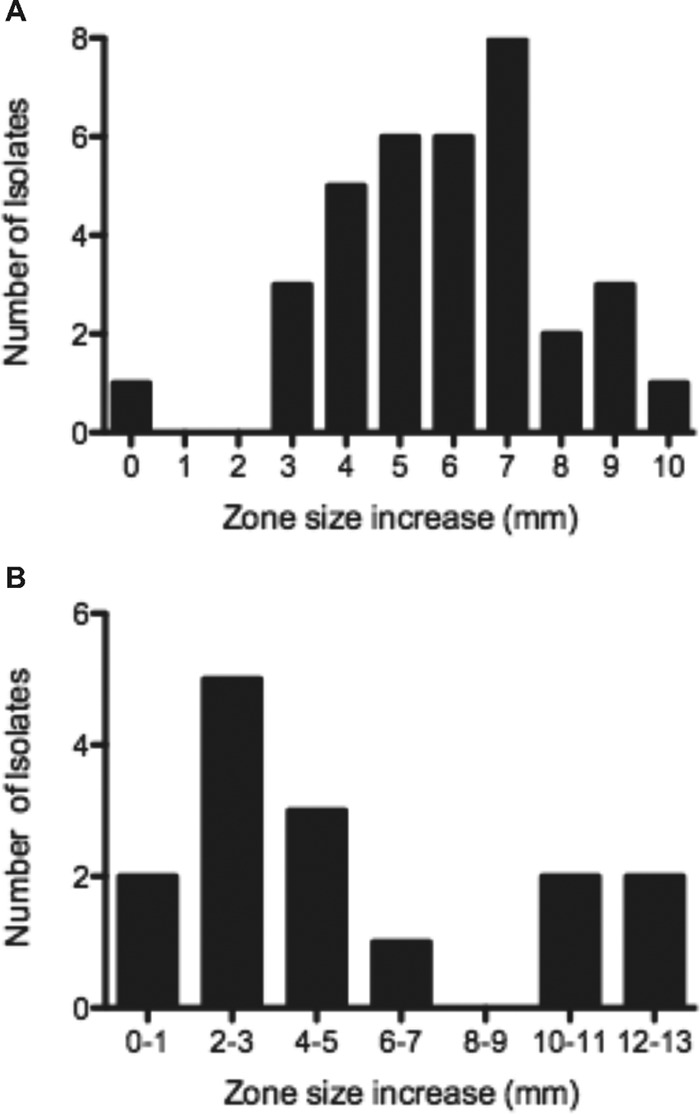
Changes in cefazolin disk diffusion zone size with cefazolin replaced at 1 h by ertapenem versus cefazolin alone for clinical bloodstream isolates of MSSA (A) or MRSA (B).

The ETP and CZ baseline susceptibilities of 35 MSSA strains were determined using CLSI broth microdilution and disk diffusion, and the relationships between the MIC and the zone of inhibition size for the two antibiotics are shown in Fig. S2 in the supplemental material. The increase in the CZ zone caused by priming for 1 h with an ETP disk prior to placing the CZ disk was measured, and the zone size increase distribution for the 35 MSSA strains is shown in Fig. 6A. We noted an increase in the zone size of ≥3 mm for 34/35 (97%) strains and no increase in the zone size for 1 strain. No strains showed a zone change of 1 or 2 mm. The mean zone size for CZ without ETP was 30.4 ± 0.9 mm, and for CZ primed with an ETP disk, it was 36 ± 0.8 mm, representing an increase in the zone size of 5.6 mm (95% confidence interval, 3.2 to 8 mm). Broth microdilution of CZ and ETP for the 15 MRSA strains showed a MIC of >8 mg/liter. The distribution of the CZ zone size increases with ETP potentiation for 15 clinical bloodstream MRSA strains is shown in Fig. 6B. An increase in zone size of ≥3 mm was seen for 10/15 (67%) strains. The mean zone size for CZ without ETP was 20.5 ± 1.2 mm, and that for CZ primed with an ETP disk was 26.3 ± 0.7 mm, representing an increase in zone size of 5.8 mm (95% confidence interval, 2.9 to 8.6 mm). The distribution, however, showed that 4 of 15 strains appeared to be hypersusceptible to the combination.

Evaluation of different beta-lactams with different penicillin binding protein (PBP) binding specificities was employed against 3 MRSA laboratory strains, 3 MSSA clinical strains, and the index isolate, rus276, using the ETP disk potentiation assay. The results are shown in Table 1 . ETP showed the greatest potentiation of CZ activity (paired *t* test; *P* = 0.026), followed by cefotaxime (*P* = 0.034). The PBP 3-selective cefaclor and PBP 4-selective cefoxitin did not show a significant increase is CZ zone size.

**TABLE 1:** Cefazolin disk diffusion potentiation assays using beta-lactams with different PBP selectivities

Strain	Zone diam (mm)^*a*^				
	CZ	ETP + CZ	CTX + CZ	CEC + CZ	FOX + CZ
rus276 (index strain)	34	40	37	37	36
MSSA1	41	41	41	41	41
MSSA2	31	35	32	31	33
MSSA3	28	32	30	30	28
MRSA MW2	25	29	26	23	26
MRSA TCH1516	21	31	25	21	26
MRSA Sanger252^*b*^	6	6	6	6	6

a CZ, cefazolin; ETP, ertapenem (PBP1); CTX, cefotaxime (PBP2); CEC, cefaclor (PBP3); FOX, cefoxitin (PBP4). Paired *t* test: CZ versus ETP plus CZ, *P* = 0.022; CZ versus CTX plus CZ, *P* = 0.033; CZ versus CEC plus CZ, *P* = 0.51; CZ versus FOX plus CZ, *P* = 0.08.

b The zone diameter (6 mm) is the diameter of the disk.

## DISCUSSION

While the therapeutic challenge of MRSA infection has been addressed with novel antibiotic development and combination therapy, the treatment of refractory MSSA bacteremia where a removable focus cannot be readily identified or addressed remains less studied. Currently, the antistaphylococcal beta-lactams (e.g., nafcillin and oxacillin) and CZ remain the preferred agents for the treatment of MSSA bacteremia and endocarditis, with daptomycin and vancomycin reserved for the highly beta-lactam-allergic patient who cannot be desensitized (30). Given the consistently inferior performance of vancomycin compared to beta-lactams in MSSA bacteremia and the availability of other newer, more potent antibiotics, the continued presence of vancomycin on this list needs serious reevaluation, and it may merit removal altogether (31).

Beta-lactams have proven to be superior antibiotics against MSSA, likely due to their dual mechanisms of action involving direct antibacterial killing and indirect potentiation of killing of S. aureus by the innate immune system (32). Despite these very useful properties, CZ or antistaphylococcal penicillin monotherapy may not be sufficient. Historically, aminoglycosides have been used in the treatment of MSSA bacteremia alongside beta-lactams, but more recent studies have demonstrated that the risk of nephrotoxicity exceeds the clinical and microbiological benefit (33). Therefore, when clinicians are faced with a case where bacteremia will not clear, the options are unknown.

Recently, combination ampicillin-plus-ceftriaxone beta-lactam therapy has been employed in the treatment of Enterococcus faecalis endocarditis in order to avoid aminoglycoside therapy (34). The rationale is the saturation of a broader spectrum of PBPs across a greater proportion of the dosing interval, resulting in more pronounced inhibition of cell wall peptidoglycan synthesis. More recently, the combined use of carbapenems plus piperacillin-tazobactam has been shown to have activity against MRSA *in vitro* and *in vivo* (35), and some older *in vitro* studies demonstrated synergy between cephalosporins and carbapenems (36). Based on these data, we were poised to utilize this strategy to clear refractory MSSA bacteremia in a case where there were no other options other than to simply wait for clearance on CZ therapy. Remarkably, the bacteremia cleared 24 h after the addition of ETP to CZ. Laboratory testing of the MSSA clinical isolate showed (i) potent synergy between ETP and CZ using traditional kill curve and checkerboard *in vitro* experiments; (ii) reduced CZ heteroresistance by ETP in a dose-dependent manner, with a more pronounced effect under high-inoculum conditions; (iii) morphological changes characterized by more potent antimicrobial activity of the combination of sub-MICs of antibiotics than with each drug alone using microscopy; (iv) synergy of ETP and CZ using PK/PD modeling at simulated drug exposures; (v) enhanced antibacterial activity of CZ plus ETP in a murine skin infection model using the clinical isolate; and (vi) reduction in biofilm formation with ETP and ETP plus CZ but an increase in biofilm formation with CZ alone at 1/4 MIC.

For reasons that are not entirely clear, biofilm formation *in vitro* has been a strong marker of persistent S. aureus bacteremia (37, 38). In this study of MSSA rus276, ETP attenuated the biofilm phenotype, whereas CZ enhanced biofilm formation. Enhanced biofilm induction with subinhibitory concentrations of various beta-lactams in S. aureus is well described, but none of the studies included carbapenems (28, 29). Whether CZ treatment failure is linked to the enhancement of biofilm formation induced by CZ and whether the enhancement of CZ pharmacotherapy by ETP is explained by these *in vitro* biofilm observations is a subject of interest for future study.

In order to determine the reproducibility of synergy between CZ and ETP against MSSA, a collection of clinical isolates was evaluated using disk diffusion. The test showed that CZ activity, measured by zone size, was increased by ETP for 34/35 (97%) MSSA strains tested.

Recent work has demonstrated synergy between meropenem and piperacillin-tazobactam against MRSA, where meropenem not only inhibits PBP 1 to complement the PBP 2 inhibition of piperacillin and the beta-lactamase inhibition of tazobactam, but also, the allosteric site binding on PBP 2A of meropenem, allowing it to be inactivated by piperacillin (35). The investigators found *in vivo* activity of this combination similar to the bacteriostatic activity of linezolid (35). We investigated the activity of CZ plus ETP against well-characterized MRSA research strains, as well as clinical isolates. ETP plus CZ showed synergy via checkerboard analysis against 2 of 3 research strains and demonstrated bacteriostatic activity against MRSA MW2 using kill curve assays. Disk diffusion showed enhancement of CZ activity by ETP for 10/15 (67%) clinical MRSA strains.

CZ has demonstrated reliable microbiological and clinical activity against MSSA over the past 4 decades, serving as a cornerstone of therapy for soft tissue infections, osteoarticular infections, bloodstream infections, and endocarditis, particularly in patients with allergies to penicillins. A recent comparison of CZ to oxacillin in the treatment of MSSA bacteremia showed similar efficacy and better tolerability of CZ (39). However, the subtle differences between antistaphylococcal penicillins and CZ that in laboratory studies appear to demonstrate less potent activity of CZ (40) may not be captured in the study of large patient cohorts. Instead, such differences may be exposed only in severe high-inoculum infections, such as MSSA infective endocarditis. Several case reports have demonstrated such failures of CZ, leading some authors and experts to regard the antistaphylococcal penicillins as “treatment for your mother” versus CZ as “treatment for your mother-in-law” (41, 42).

Two lines of scientific evidence may explain the potential pitfalls of CZ monotherapy in the treatment of MSSA endocarditis. First, some MSSA strains contain class A beta-lactamase, which hydrolyzes CZ with much greater efficiency than class B, C, and D beta-lactamases (20 times greater than cephalothin). This results in significantly reduced activity of CZ in the setting of high density and high inoculum, like endocarditis (43–46). These strains have also been linked to a reduction in function of the accessory gene regulator (47). Interestingly, the latter phenotype has been associated with persistent MRSA bloodstream infection, as well as reduced susceptibility to vancomycin, daptomycin, and cationic host defense peptides (48–51). Second, an interesting observation by some investigators has demonstrated that MSSA in the presence of neutrophils downregulates PBP 2, which happens to be the primary target of CZ (52). While CZ binds all PBPs of MSSA except PBP 4 at *C*_max_, downregulation of the PBP that is inactivated by CZ for the greatest percentage of the dosing interval would be anticipated to attenuate the activity of CZ *in vivo*.

The rationale for employing double-beta-lactam therapy with drugs with complementary PBP binding profiles is that it would maximize the potency of anti-staphylococcal therapy for a greater percentage of the dosing interval. The synergy would be beneficial, not only between the administered antibiotics, but also with innate cationic host defense peptides. In addition, potential downregulation of the primary CZ PBP 2 target could be circumvented by targeting an alternative PBP, such as PBP 1, by ETP.

We confirmed prior findings (35) that indeed two beta-lactam drugs that alone would have no demonstrable activity against MRSA demonstrated bacteriostatic activity when used together. The prior study focused on meropenem and piperacillin-tazobactam, while this study evaluated ETP and CZ. ETP and CZ would be considerably more favorable to coadminister, not only because of simpler dosing schedules, but also because of their narrower antibacterial spectrum. Nevertheless, while the CZ-plus-ETP combination may be readily applied to patients with refractory MSSA infection as a way to enhance CZ monotherapy, much more rigorous evaluation at the laboratory and clinical levels of double-beta-lactam therapy against MRSA infection is needed.

In conclusion, this study demonstrated synergy between CZ and ETP against MSSA, using both *in vitro* and *in vivo* laboratory methods. Synergy between CZ and ETP against some MRSA strains was also noted. The application of double-beta-lactam therapy by adding ETP to CZ may provide clinicians an option in treating refractory MSSA bacteremia where clearance cannot be achieved by removing an obvious focus, such as a catheter, abscess, or vegetation. These cases may be due to MSSA strains that produce type A beta-lactamase, which more efficiently hydrolyzes cefazolin. This notion is supported by preliminary data in our laboratory showing that the synergy of ETP and CZ is more pronounced in MSSA with type A beta-lactamase. In addition, the greater benefit of ETP in protecting the activity of CZ under high-inoculum conditions (shown in the population analysis studies) suggests that ETP may be important in cases where CZ is at high risk of failure due to enhanced beta-lactamase hydrolysis, due to either inoculum size and/or a class A beta-lactamase in MSSA. Future studies of this drug combination will continue to focus on the effect of beta-lactamase types in MSSA and of the bacterial inoculum on the magnitude of the synergistic effect between ETP and CZ, as well as studying the effects of various beta-lactamase inhibitors on ETP-CZ synergy.

## Supplementary Material

Supplemental material
