# Supplementary material for: Cefazolin and Ertapenem, a Synergistic Combination Used To Clear Persistent Staphylococcus aureus Bacteremia
Source: Antimicrob Agents Chemother. 2016 Oct 21;60(11):6609–18. doi: 10.1128/AAC.01192-16 (PMC5075066; doi:10.1128/AAC.01192-16)
Supplement: Supplemental material [file supp_60_11_6609__index.html]

Supplemental material 

# Cefazolin and Ertapenem, a Synergistic Combination Used To Clear Persistent Staphylococcus aureus Bacteremia

## Supplemental material

- Supplemental file 1 -

  Figures S1 and S2

  PDF, 73K
