# Supplementary material for: Cefazolin and Ertapenem, a Synergistic Combination Used To Clear Persistent Staphylococcus aureus Bacteremia
Source: Antimicrob Agents Chemother. 2016 Oct 21;60(11):6609–18. doi: 10.1128/AAC.01192-16 (PMC5075066; doi:10.1128/AAC.01192-16)

Supplemental Figure 1. Assessment of biofilm production of MSSA rus276 in subinhibitory concentrations of ETP, CZ, or both drugs. Results are expressed as ratio of crystal violet absorbance (OD_{570}) to bacterial density (OD_{600}) to adjust for growth differences in the different conditions (*$p<0.05$ vs control).

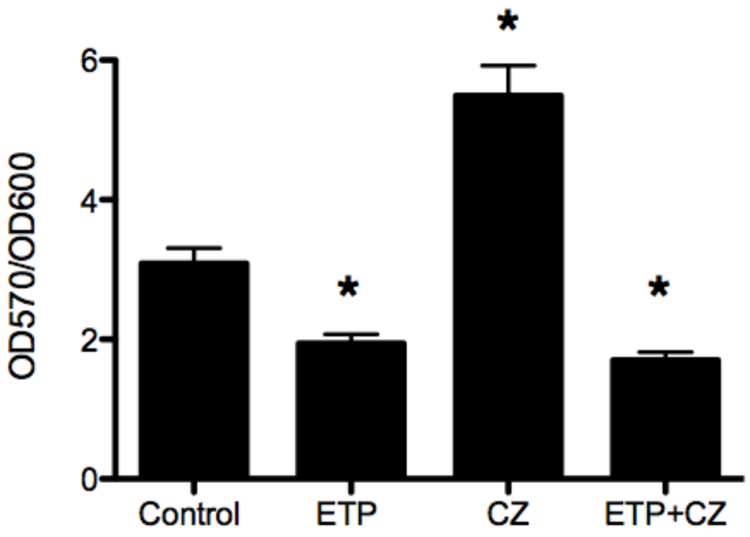

Supplemental Figure 2. Relationship between zone size by disk diffusion and broth microdilution of MSSA isolates in this study.

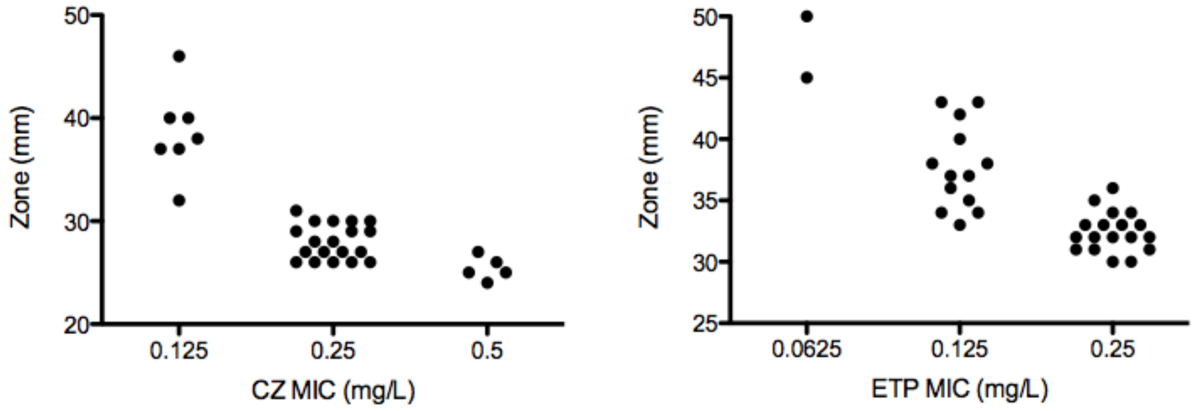

Supplement: Supplemental material [file AAC.01192-16_zac011165678so1.pdf]
